# Factors Influencing Public Awareness of and Attitudes Toward Palliative Care: A Cross-Sectional Analysis of the 2018 HINTS Data

**DOI:** 10.3389/fpubh.2022.816023

**Published:** 2022-04-07

**Authors:** Xinyu Lu, Jiawei Liu

**Affiliations:** ^1^School of Journalism and Communication, Shanghai International Studies University, Shanghai, China; ^2^School of Journalism and Communication, Jinan University, Guangzhou, China

**Keywords:** palliative care, awareness, attitudes, HINTS, survey

## Abstract

**Background:**

The global burden of serious health-related suffering requiring palliative care has been projected to grow significantly by 2060, which indicates the imminent need for integrating palliative care into health systems globally. Moreover, research evidence has been accumulating in support of the earlier adoption of palliative care into the treatment course of serious life-threatening illnesses. However, barriers to earlier access to palliative care still remain, which might be attributable to the global lack of awareness of palliative care and the prevalence of negative perceptions and attitudes. To address this, further investigation of the influencing factors of public perceptions of palliative care is imperative to help inform and develop effective targeted public health campaigns and education messages aimed at improving views of palliative care and thereby early access.

**Methods:**

We used data from the Health Information National Trends Survey (HINTS), a nationally representative cross-sectional survey routinely administrated by the National Cancer Institute from the United States. Specifically, we analyzed the latest palliative care data from HINTS 5 Cycle 2 data set. Sociodemographic characteristics, individual factors such as self-perceived health status, and interpersonal factors such as relationship quality were examined as predictors of public awareness of and attitudes toward palliative care. Survey data were analyzed using SPSS 26 with multiple hierarchical regression tests.

**Results:**

Results showed that people's quality of interpersonal relationships was a significant influencing factor of their awareness of and attitudes toward palliative care. Moreover, cancer diagnosis history and perceived healthcare quality were found to jointly affect their awareness of palliative care; perceived health status and patient centeredness interacted to influence their awareness of and attitudes toward palliative care. Finally, female, non-white, and poorer people were more aware of palliative care, while female and more educated people had more favorable attitudes.

**Conclusions:**

The quality of social relationships emerges as a significant predictor of people's awareness of and attitude toward palliative care, as treatment options and decisions of serious life-threatening illnesses often involve the patients' family. The results hold strong implications for public health campaigns and education messages aiming at changing people's views of palliative care, which ultimately improve end-of-life outcomes.

## Introduction

The global population is aging at an unprecedented rate, coupled with the rise of patients diagnosed with chronic and life-limiting illnesses (1). Palliative care, specialized medical care to relieve suffering and optimize quality of life for people living with a serious illness, has been deemed increasingly crucial by global public health and medical professionals and researchers [e.g., (2–4)]. The global burden of serious health-related suffering requiring palliative care has been projected to grow significantly by 2060, which indicates the imminent need for integrating palliative care into health systems globally (5). Moreover, research evidence has been accumulating in support of the earlier adoption of palliative care into the treatment course of serious life-threatening and life-limiting illnesses (6–10).

Despite this call for earlier access to palliative care in the illness course, barriers to using it remain. It is predicted that only about 14% of patients worldwide who need it receive it (11). This might be attributable to the global lack of awareness and knowledge of palliative care among the general public [for a complete review, see (12)], particularly about the benefits it can offer to patients. The prevalence of negative beliefs and attitudes toward palliative care has been deemed as another major hurdle for palliative care access [e.g., (13–15)]. Typical negative beliefs held by patients and family caregivers regarding palliative care are negative connotations of deaths [e.g., (16, 17)] and associations of palliative care acceptance with giving up hope [e.g., (13, 14)]. Given that extant research suggests the vital role of palliative care awareness (18, 19) and negative attitudes toward palliative care (18, 20) in influencing people's willingness to accept palliative care, understanding what might affect and enhance the general public's awareness of and attitudes toward palliative care has become more critical than ever for public health and medical researchers and professionals.

Extant literature on awareness of and attitudes toward palliative care are mainly descriptive, outlining health care professionals and the publics' awareness, attitudes and perceptions toward palliative care with mixed results [e.g., (12, 21–25)]. While some research found that most people had positive attitudes and good knowledge of palliative care (24, 25), other studies found the opposite (12, 21, 23, 26). Thus, it is important to address the prior inconsistent findings and further examine public awareness of and attitudes toward palliative care.

Unlike the wealth of research delineating people's level of awareness of and attitudes toward palliative care above, research examining its predictors is quite limited and has identified important individual or intrinsic factors such as caregiver experience and age (27), as well as awareness of hospices (23). For example, McLachlan and Philip (23) found that people who were aware of hospices had more favorable attitudes toward palliative care. In addition, research on predictors of people's awareness of and attitudes toward diseases and treatments in general has yielded meaningful implications and suggested the important role of other intrinsic, personal predictors such as perceived health status (28), self-care confidence (29) and past health care appointment (30), and extrinsic, interpersonal factors such as family support (31, 32) and patient-doctor relationship (32, 33) in people's understanding of the diseases and thereby treatment adherence. Accordingly, it remains to be examined whether the influence of such individual and interpersonal factors would extend to the palliative care context.

These possibilities could be explained by the social ecological model, which serves as this study's theoretical framework. According to the social ecological model proposed by Bronfenbrenner (34), the five main levels of influence for health-related behaviors and conditions are intrapersonal or individual factors (i.e., awareness, attitude, behavioral intention), interpersonal/network factors (e.g., social support from family members, coworkers, friends, and health professionals), community factors (i.e., relationships among organizations/institutions), and public policy factors (e.g., local, state, and national law). This model suggests that health-related behavior is a property of the coupled individual-group-environment system, and cannot in general be properly attributed to one subsystem in isolation from others (34). It has been widely used in the health promotion field to explain a patient's adherence to a medication regimen (35–38). Since treatment options and decisions of serious life-threatening and life-limiting illnesses often involve the patients' social environment, particularly their relatives (i.e., family caregivers), interpersonal factors also play a prominent role in the decision-making processes. Therefore, further work examining personal and interpersonal factors influencing the general public's awareness of and attitudes toward palliative care from a social ecological theoretical stance is needed.

Given the much-documented positive effects of earlier access to palliative care in the disease treatment process [e.g., (9, 10)], further investigation of the influencing factors of the general public's perceptions of palliative care is imperative to help inform and develop effective public health campaigns and palliative care education messages aimed at improving perceptions and views of palliative care and thereby early access. Thus, drawing on the social ecological model, this study examined how both individual-level factors (perceived health status, perceived self-care ability, and perceived healthcare quality) and interpersonal-level factors (interpersonal relationship quality and past patient-centered communication experiences) affect the general public's awareness of and attitude toward palliative care.

## Methods

### Data

This study used publicly available data from the Health Information National Trends Survey (HINTS), a nationally representative cross-sectional survey routinely administrated by the National Cancer Institute. The HINTS survey aims to track American public's awareness of, attitudes toward, and the actual use of cancer- and health-related information (https://hints.cancer.gov/). Specifically, this study analyzed the latest palliative care data from HINTS—HINTS 5 Cycle 2 data set [response rate = 32.9%, *N* = 3,504; (39)], which was collected between January and May of 2018. Survey data were collected by mail, with a $2 monetary incentive to encourage participation. A detailed description about HINTS methodology can be found elsewhere (40).

Given that the purpose of this study is to examine the public's awareness of and attitudes toward palliative care, the data analysis excluded those who have never heard of palliative care. Specifically, 65.2% (*n* = 2,283) of the sample answered “I have never heard of it” to the question “How would you describe your level of knowledge about palliative care?”), and were not asked follow-up palliative care-related questions, such as awareness of the goal of palliative care and negative attitudes toward palliative care. Respondents who answered one of the other two responses (*n* = 1,162)—“I know a little bit about palliative care” (20.3%) or “I know what palliative care is and could explain it to someone else” (12.8%)—answered follow-up questions and were included for further analysis. In addition, since one of the predictor variables this study examined was the quality of people's past patient-centered communication experiences, those participants who had not visited doctors, nurse, or other health professional within the past 12 months were excluded from the data analysis. Thus, the final sample for the data analysis was 1,031.

### Measurements

HINTS survey questionnaires were constructed based on measurements from previous national-level surveys, such as CDC's Behavioral Risk Factor Surveillance System, and smaller-scale health-related surveys, or created by the HINTS program research team at the National Cancer Institute.

*Perceived health status* was measured by one item, adopted from the 12-Item Short Form Health Survey (SF-12) (41). Respondents were asked: “In general, would you say your health is?.” Responses were scored on a five-point scale (5 = poor to 1 = excellent) and were reversely coded later (*M* = 3.61, *SD* = 0.94).

*Perceived self-care ability* was measured by one item, which asked: “Overall, how confident are you about your ability to take good care of your health?.” Responses were scored on a five-point Likert scale (5 = not confident at all to 1 = complete confident) and were reversely coded later (*M* = 4.03, *SD* = 0.77).

*Perceived healthcare quality* was measured by a single item, adopted from the Cancer Care Outcomes Research and Surveillance (CANCors) patient survey. Respondents were asked to rate the quality of health care they received in the past 12 months using a five-point Likert scale (5 = *poor* to 1 = *excellent*), which were reversely coded later (*M* = 4.15, *SD* = 0.88).

*Quality of interpersonal relationships* was measured by constructing six items that asked whether respondents had: (1) anyone they can count on to provide them with emotional support when they need it—such as talking over problems or helping them make difficult decisions, (2) friends or family members that they talked to about their health, (3) someone to prepare their meals if they were unable to do it themselves, (4) someone to take them to the doctor if they need it, (5) someone to help with their daily chores if they were sick, and (6) someone to run errands if they needed it. Respondents answered using a five-point Likert scale (1 = *never* to 5 = *always*), which was later averaged (*M* = 4.11, *SD* = 1.0, Cronbach's alpha = 0.91).

*The quality of past patient-centered communication experiences* was measured by seven items, revised from the Consumer Assessment of Health Plans Study (CAHPS^®^) 2.0's Adult Core Survey and Adult Supplemental Questions. Respondents were asked to rate how often their doctors, nurses, or other health professionals they saw during the past 12 months: (1) gave them the chance to ask all the health-related questions they had, (2) gave the attention they needed to their feelings and emotions, (3) involved them in decisions about their health care as much as they wanted, (4) made sure they understood the things they needed to do to take care of their health, (5) explained things in a way they could understand, (6) spent enough time with them, and (7) helped them deal with feelings of uncertainty about their health or health care. Respondents answered using a four-point scale (4 = *never* to 1 = *always*), which was later reversely coded and then averaged (*M* = 3.42, *SD* = 0.61, Cronbach's alpha = 0.92).

*Awareness of the goal of palliative care* was measured using four items. Respondents were asked to rate their agreement with four positive statements about the goal of palliative care: (1) to help friends and family to cope with a patient's illness, (2) to offer social and emotional support, (3) to manage pain and other physical symptoms, and (4) to give patients more time at the end of life. Respondents answered using a four-point Likert scale (1 = *strongly agree* to 4 = *strongly disagree*), which was later reversely coded and then averaged (*M* = 3.42, *SD* = 0.50, Cronbach's alpha = 0.87). Higher score indicated greater awareness toward palliative care.

*Attitude toward palliative care* was constructed using two items. Respondents were asked to rate their agreement with two negative statements about palliative care: (1) Accepting palliative care means giving up, and (2) when I think of palliative care, I automatically think if deaths. Responses were scored on a 4-point Likert scale (1 = *strongly agree* to 4 = *strongly disagree*), which was later reversely coded and then averaged (*M* = 1.87, *SD* = 0.75; *r* =.38, *p* < 0.001). Higher score indicated less favorable attitude toward palliative care.

*Cancer diagnosis* was included as a moderating variable in this analysis as the predictors of people's awareness and attitude toward palliative care might differ between people with or without cancer history. It was measured by one item, revised from National Health Interview Survey (NHIS) 2000's Adult Core Questionnaire (42). A single question was asked: “Have you ever been diagnosed as having cancer?”

Demographics were included as control variables in this analysis. Included were age, gender (0 = female and 1 = male), marital status (0 = unmarried and 1 = married), education (ranging from 1 = less than high school to 4 = college graduate or higher), household income, and race (0 = non-white and 1 = white).

## Results

The average age of the sample was 57 years, 30.7% were male, and 61.3% of the sample had at least a college degree. 54.9% were married or living as married; 67% had annual household incomes above $50,000; 84.5% were Non-Hispanic White; 12.9% were African American, and 8% were Hispanics. In terms of knowledge of palliative care, a majority of the respondents (61.6%) self-reported that they knew a little bit about palliative care, followed by the respondents who knew what palliative care was and could explain it to someone else (38.4%). The frequency of visits with doctors, nurses, or other health professional within the past 12 months ranged from 1 (17.5%) to 10 or more times (9.3%), with the median of 3 times. 19% of the sample had been diagnosed with cancer.

Two separate hierarchical regression analyses were conducted, each of which predicting one of the two dependent variables (awareness of the goal of palliative care and attitudes toward palliative care). The predictor variables, control variables, interaction terms were entered in the following manner: (1) in the first block, demographic variables were entered as control variables; (2) the personal (*perceived health status, perceived self-care ability*, and *perceived healthcare quality*) and interpersonal (*quality of interpersonal relationships* and *the quality of past patient-centered communication experiences*) influencing factors were entered in the second block as the main predictor variables (after being mean centered); and (3) seven interaction terms were created by multiplying the centered moderator and centered main predictors.

First, Table 1 presents the hierarchical regression model results explaining predictors of people' awareness of the goal of palliative care. In terms of demographics, gender, household income and race correlated with people's awareness of the goal of palliative care. Specifically, being female, non-white, and poorer people, increased respondents' levels of awareness. However, age, education, and marital status were not significantly related with people's awareness of the goal of palliative care (see Table 1).

**Table 1:** Hierarchical regression model predicting awareness of the goal of palliative care.

**Block 1: control variables**	**Model 1 β**	**Model 2** **β**	**Model 3 β**
Age	−0.001	−0.001	0.010
Gender	−0.076^*^	−0.077^*^	−0.084^*^
Education	−0.008	−0.016	−0.011
Income	−0.095^*^	−0.112^*^	−0.090
Race	−0.095^*^	−0.089^*^	−0.087^*^
Marital status	0.065	0.023	0.005
**Block 2: Main predictors**	**Model 1**	**Model 2**	**Model 3**
Quality of relationships (QR)	-	0.093^*^	0.098^*^
Patient centeredness (PC)	-	0.045	0.072
Quality of healthcare (QH)	-	0.026	−0.026
Perceived health (PH)	-	−0.039	−0.059
Own ability to take care (OA)	-	0.071	0.060
**Block 3: Interaction terms**			
Cancer diagnosis (CA)^*^ QR	-	-	−0.048
CA^*^PC	-	-	−0.030
CA^*^QH	-	-	0.120^*^
CA^*^PH	-	-	0.004
CA^*^OA	-	-	0.034
PH^*^QR	-	-	0.115^**^
PH^*^PC	-	-	−0.080^*^
R^2^	2.3%	4.3%	6.6%
Adjusted R^2^	1.5%	2.8%	4.2%
Overall F	2.882	2.915	2.780
Df	6, 721	5, 716	7, 709
F Change	-	2.908	2.509
R^2^ Change	-	1.9%^*^	2.3%^*^

a *p < 0.05.*

** *p < 0.01*.

Regarding the main predictors of awareness of palliative care, the results only indicated a significant positive relationship between people's quality of interpersonal relationships and their levels of awareness about palliative care (β = 0.093, *p* < 0.05). In other words, respondents who had more stable interpersonal relationship, i.e., having friends or family to talk to, provide emotional and physical support, were more likely to believe that the goal of palliative care was to offer aid and support to patients. This relationship was significant after controlling for all of the control variables. In addition, people's awareness of the goal of palliative care were not found to be significantly associated with their perceived health status (β = −0.039, *p* = 0.41), perceived self-care ability (β = 0.071, *p* = 0.12), the quality of healthcare received (β = 0.026, *p* = 0.65), and the quality of their past patient-centered communication experiences (β = 0.045, *p* = 0.41). This indicated a stronger effect of interpersonal factors, compared to personal factors, on people's perceptions of the goal of palliative care. The main predictor variables totally explained 4.3% of the variance in the outcome variable.

**Figure 1:**
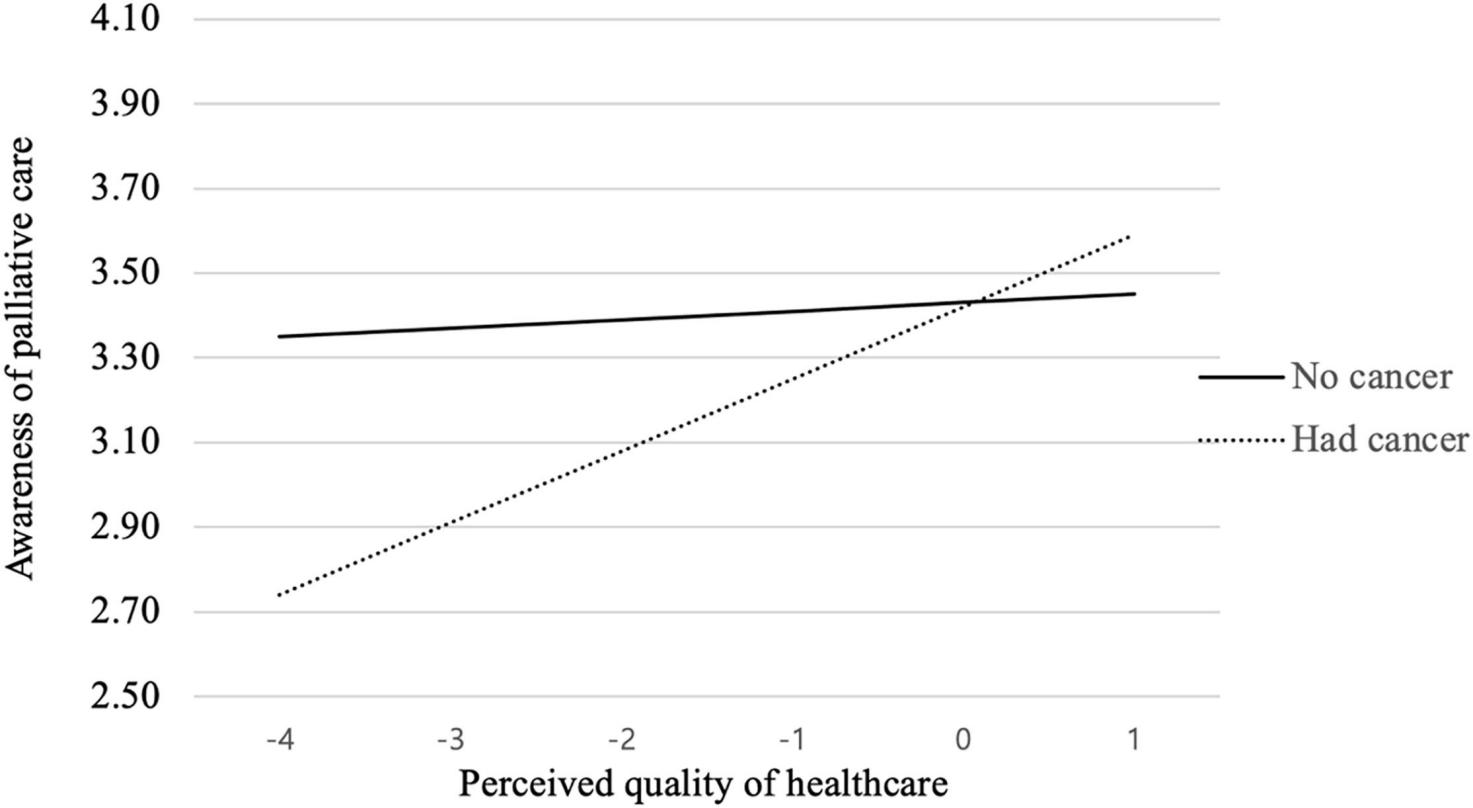
*Cancer history* × *Quality of healthcare* interaction on awareness of palliative care.

As Table 1 presents, the interaction term between cancer diagnosis and the perceived quality of healthcare they received in the past 12 months had a statistically significant correlation with people's awareness of the goal of palliative care (β = 0.12, *p* < 0.05). To more be specific, as shown in the interaction graph (see Figure 1), for people who had been diagnosed with cancer before, quality of healthcare was positively associated with their awareness of palliative care, while people with no cancer history displayed no such tendency. Besides this, cancer diagnosis was not found to moderate the influence of other personal and interpersonal factors on people's awareness of the goal of palliative care.

**Figure 2:**
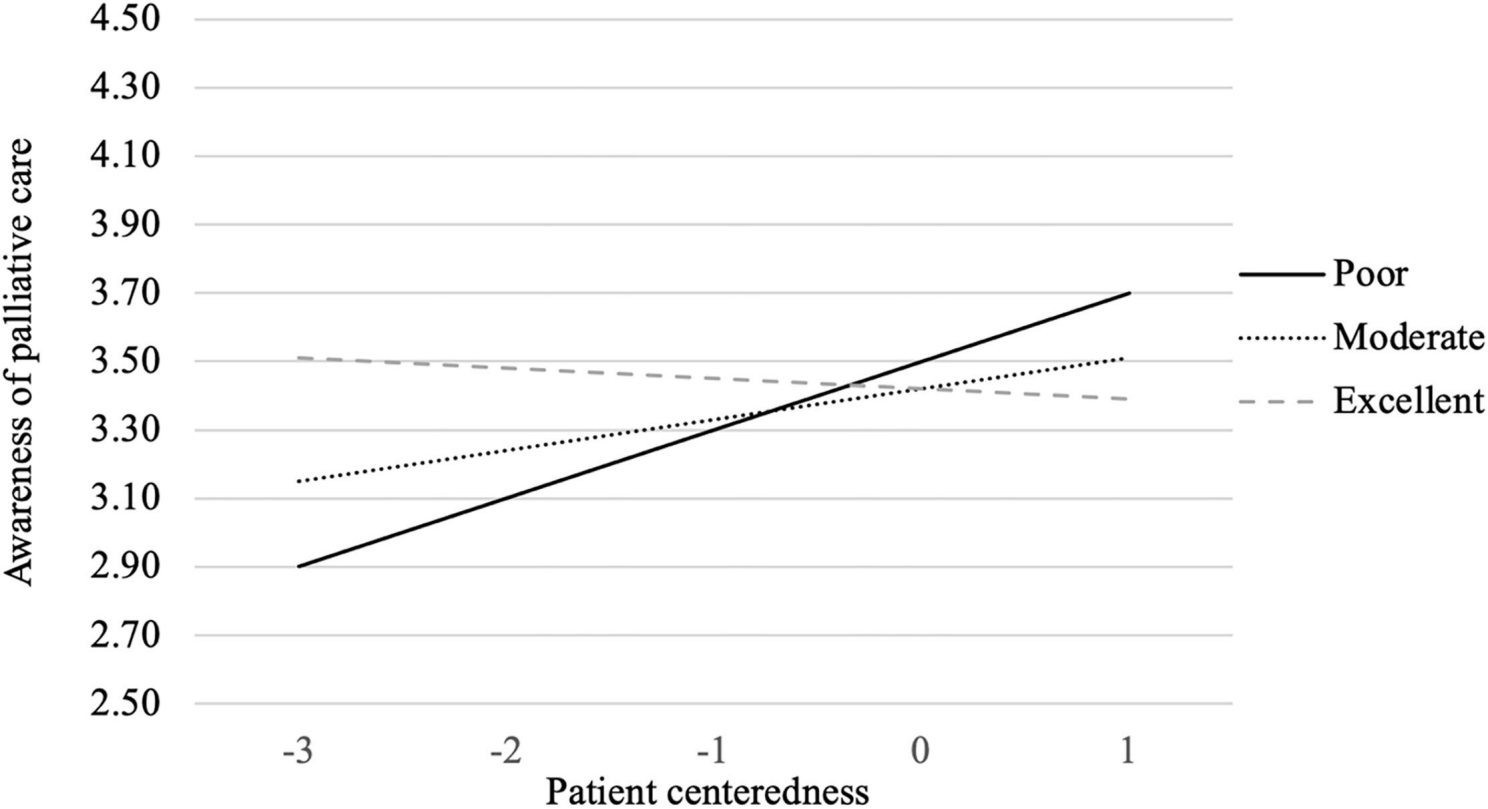
*Perceived health status* × *Patient centeredness* interaction on awareness of palliative care.

As shown in Table 1, the interaction between perceived heath status and patient centeredness had a significant relationship with people's awareness of the goal of palliative care (β = 0.12, *p* < 0.01). To better illustrate the interaction effects, perceived health status was further divided into three groups based on mean ± 1SD (i.e., poor, moderate or excellent perceived health condition). As the interaction graph illustrates (see Figure 2), for people who think they are in excellent health condition, patient centeredness was not playing an influential role in influencing their awareness of the goal of palliative care. However, for people who think they are in poor or moderate health condition, the level of perceived patient centeredness is positively related with their awareness of palliative care.

**Figure 3:**
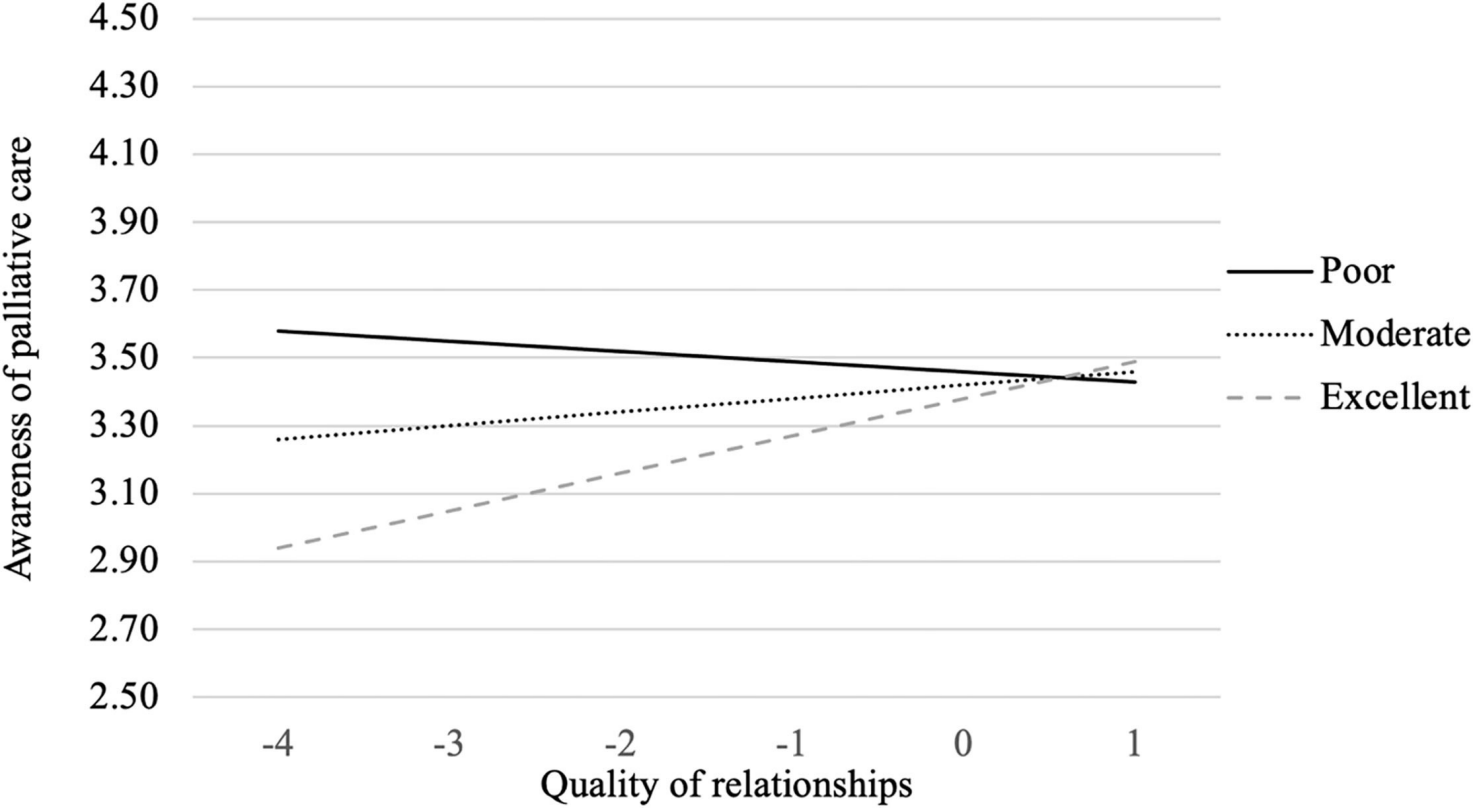
*Perceived health status* × *Quality of interpersonal relationships* interaction on awareness of palliative care.

In addition, the interaction between perceived heath status and the quality of interpersonal relationships had a significant relationship with people's awareness of the goal of palliative care (β = −0.08, *p* < 0.05). As the interaction graph illustrates (see Figure 3), people reporting better quality of interpersonal relationship were more likely to have higher awareness of palliative care when perceiving themselves in better health condition. The more stable interpersonal relationship they have, the higher awareness of palliative care's goal to offer aid and support to patients. However, the opposite holds true for people who thought they were in poor health condition.

Table 2 presents the second hierarchical regression model results explaining predictors of people' attitudes toward palliative care. In terms of demographics, gender and education correlated with people's attitudes toward palliative care. Specifically, female and more educated people are more likely to have positive attitudes toward palliative care, while age, income, race, and marital status were not significantly related (see Table 2).

**Table 2:** Hierarchical regression model predicting attitudes toward palliative care.

**Block 1: control variables**	**Model 1 β**	**Model 2** **β**	**Model 3 β**
Age	0.051	0.051	0.151
Gender	0.087^*^	0.083^*^	0.082^*^
Education	−0.146^*^	−0.118^*^	−0.123^*^
Income	0.023	0.062	0.064
Race	−0.002	0.007	0.006
Marital status	−0.016	0.010	0.015
**Block 2: Main predictors**	**Model 1**	**Model 2**	**Model 3**
Quality of relationships (QR)	-	−0.071	−0.088^*^
Patient centeredness (PC)	-	0.054	0.053
Quality of healthcare (QH)	-	−0.083	−0.106
Perceived health (PH)	-	−0.046	−0.030
Own ability to take care (OA)	-	−0.078	−0.087
**Block 3: Interaction terms**			
Cancer diagnosis (CA)^*^ QR	-	-	0.035
CA^*^PC	-	-	0.056
CA^*^QH	-	-	0.013
CA^*^PH	-	-	−0.004
CA^*^OA	-	-	−0.002
PH^*^QR	-	-	−0.025
PH^*^PC	-	-	−0.078^*^
R^2^	3.3%	5.5%	6.8%
Adjusted R^2^	2.5%	4.2%	4.7%
Overall F	4.387	4.120	3.133
Df	6, 780	5, 775	7, 768
F Change	-	3.708	1.551
R^2^ Change	-	2.3%^*^	1.3%

* *p < 0.05*.

Regarding the influencing factors of people's attitudes toward palliative care, the results only indicated a significant negative relationship between people's quality of interpersonal relationships and their negative attitude toward palliative care (β = 0.093, *p* < 0.05). In other words, respondents who had more stable interpersonal relationship, i.e., having friends or family to talk to, provide emotional and physical support, were less likely to associate palliative care with giving up and death. This relationship was significant after controlling for all of the control variables. In addition, people's attitudes toward palliative care were not found to be significantly associated with their perceived health status (β = −0.046, *p* = 0.32), perceived ability to take care of their health (β = −0.078, *p* = 0.07), the quality of healthcare received (β = −0.083, *p* = 0.13), and the quality of their past patient-centered communication experiences (β = 0.054, *p* = 0.30). This indicated a stronger effect of interpersonal factors, compared to personal factors, on people's negative perceptions of palliative care. The main predictor variables totally explained 5.5% of the variance in the outcome variable.

**Figure 4:**
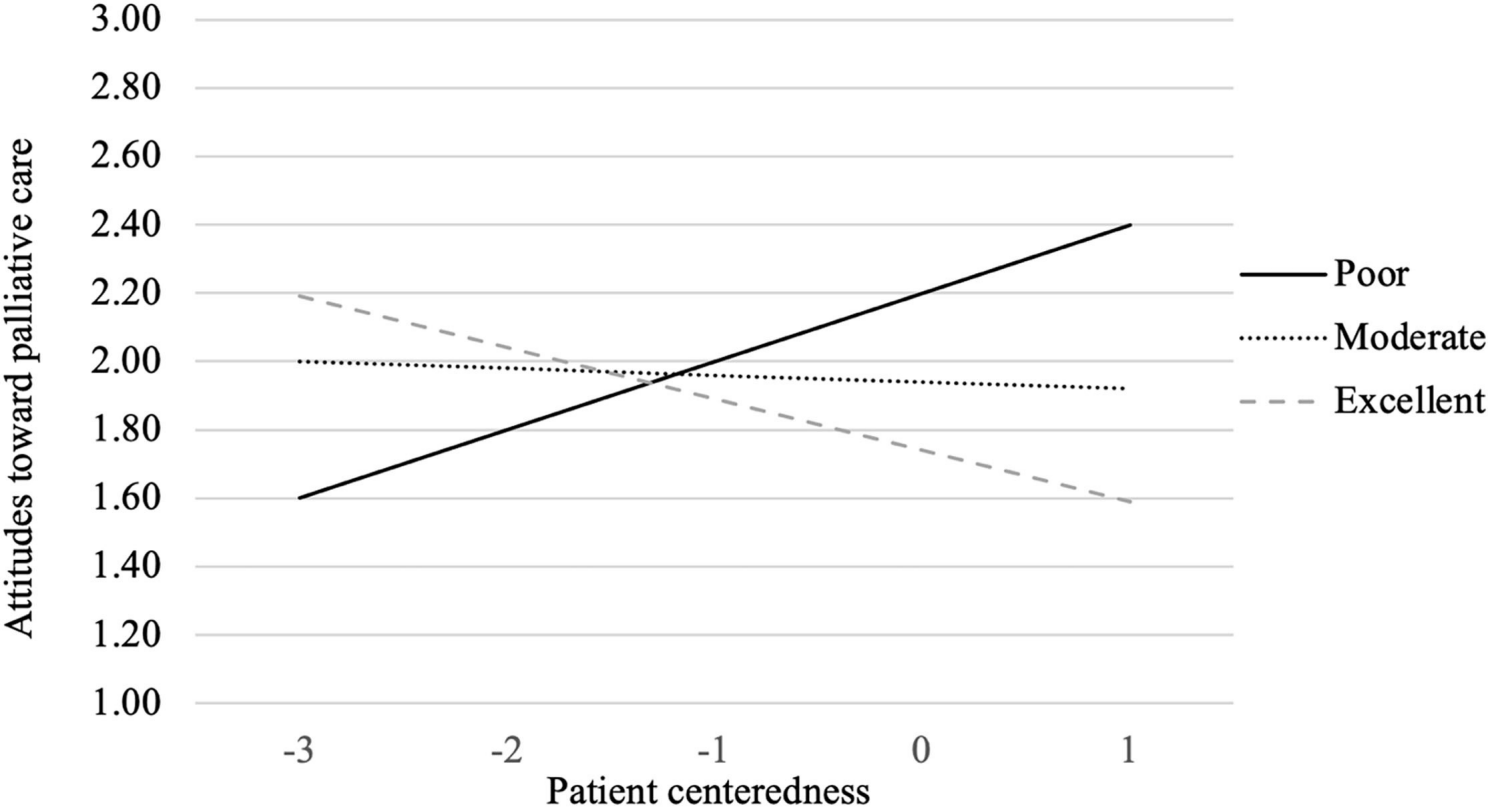
*Perceived health status* × *Patient centeredness* interaction on attitude toward palliative care.

In addition, as Table 2 presents, cancer diagnosis was not found to moderate the influence of all the predictor factors on people's attitude toward palliative care. Only the interaction between perceived heath status and patient centeredness had a significant relationship with people's attitudes toward palliative care (β = −0.078, *p* < 0.05). Similarly, to better illustrate the interaction effects, perceived health status was further divided into three groups based on mean ± 1SD (i.e., poor, moderate or excellent perceived health condition). As the interaction graph illustrates (see Figure 4), for people who think they are in poor health condition, patient centeredness is associated with less favorable attitude toward palliative care (i.e., associating palliative care with giving up and death). However, for people who think they are in moderate or excellent health condition, the level of perceived patient centeredness is associated with more favorable attitude toward palliative care.

## Discussion

Palliative care aims to provide both physical and emotional relief for patients and their caregivers. Yet, barriers to palliative care consultation still exist. Factors such as awareness of and attitude toward palliative care have been identified as key facilitators of enrollment in palliative care (18–20). Thus, it is important to figure out what influencing factors are associated with people's awareness of and attitudes toward palliative care. Drawing on the social ecological model (34), this study examined what individual and social factors play a critical role in influencing people's awareness of and attitudes toward palliative care.

The study results revealed several significant insights. First, according to the nationally representative HINTS survey collected in the U.S., the majority of the respondents had never heard of palliative care, while only a small proportion could explain what palliative is. Similar to previous studies (12, 21, 23, 26), this finding suggests a lack of education and public knowledge about palliative care. Given the well-documented benefits of early adoption of palliative care in the illness trajectory [e.g., (2, 6, 7)], this indicates the necessity of clear policies and educational programs that informs the publics of what palliative care really is and its goal (43).

Second, our results indicated that demographic variables, including gender, household income, education, and race, were associated with people's awareness of the goal of palliative care and attitudes. In particular, we found out that female, non-white, and poorer respondents were more likely to have greater awareness of the goal of palliative care. In addition, female, more educated respondents reported more favorable attitude toward palliative care. This finding provides a more nuanced understanding of the perceptions of palliative care among various demographics, as prior literature was inconclusive and contradictory on this. While one study found supporting evidence of a positive link between age and access to palliative care (44), another failed to find this pattern (13). This study also found no significant difference in terms of awareness and attitudes toward palliative care associated with age. In terms of gender, in line with the prior finding that females had more knowledge of palliative care [e.g., (19)] and had more access to palliative care than males (13), this study found that women were more likely to have greater awareness of palliative care and more favorable attitudes compared to men. The findings are consistent with previous research, which suggests that women exhibit stronger health-seeking behaviors in response to both physical and mental health concerns than men (45–47). While the role of race was inconsistent in prior research [e.g., (2, 13, 14)], this study found that non-white had greater awareness of the goal of palliative care, which is similar to the finding that Black adult patients with advanced cancer were more likely to have received palliative care consultation than non-Hispanic White patients (48). This might be attributable to their higher symptom burden, which naturally demands the assistance of palliative care services to managing their symptoms (2).

This study also found significant differences in awareness of palliative care by household income, with poorer people reporting greater awareness. The expensive costs of curative treatment for those life-threatening and life-limiting illnesses, coupled with low survival rates (49), may be a possible explanation for this. Given the relative cheaper costs of palliative care [e.g., (49, 50)], people with lower income might be more willing to seek out and learn about this alternative option, while those richer may prefer to continue receiving active life-prolonging curative treatment regardless of the prognosis. Additionally, this study found disparities in attitudes toward palliative care by education level. In contrast to one study by Collins et al. (27) which found no significant relationship between education and attitudes to palliative care, this study found a positive link between education level and favorable attitude toward palliative care. People with higher education level are likely to have higher general health literacy, which may extend to the palliative care context.

Such insights are meaningful for equitably improving the awareness and attitudes toward palliative care among all these groups. Informative and effective educational programs and referral to palliative care services should be developed to increase all the populations' knowledge and attitudes toward palliative care. Thus, health practitioners and researchers are suggested to develop targeted message interventions to increase greater adoption of palliative care based on different demographic segments. It is crucial to help people with lower awareness of and less favorable attitude toward palliative care better understand the goal of palliative care and its benefits. For example, health professionals are suggested to inform patients, particularly those richer and white people, that palliative care is not the same as end-of-life care and can be performed alongside curative treatment for any patient living with a serious illness.

Third, compared to personal factors, interpersonal ones were found to play a more prominent role in influencing people's awareness of and attitude toward palliative care. In general, individuals who had more stable interpersonal relationship, i.e., having friends or family to talk to, and providing them with emotional and physical support, showed greater awareness of the actual goal of palliative care and reported more favorable attitude. For patients who had more stable interpersonal relationships, their family or friends tend to provide them with strong emotional and physical support and might be more willing to learn how to improve quality of life for them, thereby fostering the perception of a more supportive social environment. Accordingly, patients and their caregivers might be more willing to hear about, discuss and have positive perceptions of the palliative care treatment option, which focuses on improving quality of life by managing the emotional, physical and spiritual effects of serious illnesses (51). Since patients with life-threatening and life-limiting illnesses often rely on their caregivers for treatment options and decisions, higher level of perceived social support yielded from stable interpersonal relationships fosters their willingness to engage with palliative care, hence higher awareness and attitudes toward palliative care. This finding is consistent with the ecological model (34), confirming the primary role of interpersonal-level factors in explaining people's awareness and attitudes toward palliative care. The finding is also in line with prior research suggesting that the perception of a supportive social environment helps provide a beneficial context for chronic illness self-management (52). Therefore, future educational programs aiming to improve the general public's awareness of and attitudes toward palliative care should focus on enhancing people's perception of a supportive social environment, as one of the goals of palliative care is to relieve physical, psychological, social, and spiritual sufferings among patients and caregivers *via* building a supportive environment.

One interesting finding worth noting is that for people who perceived themselves in worse or poor health condition, the quality of interpersonal relationships was surprisingly negatively related to awareness of the goal of palliative care. Those who perceived themselves in poor health condition might be in stronger need of emotional and physical support from their family and friends. When such needs were not satisfied (i.e., less stable interpersonal relationships), they were more likely to seek out alternative ways to offer emotional and physical support and might be more willing to learn about the palliative care option, thereby being more aware of its goal. Instead, for those who received strong emotional and physical support from their family and friends, they tend to be more reliant on them and may be less likely to adopt palliative care. This finding is particularly meaningful since it suggests the importance of emphasizing emotional and physical supports that could be offered by palliative care when designing educational messages aiming at improving patients' awareness of palliative care. For example, it is important to clarify the misconception that palliative care is only provided in hospital and hastens death. Instead, palliative care can help patients stay safely at home with their families (53).

Fourth, though we did not find significant main effects of personal factors on people's awareness and attitude toward palliative care, results demonstrated several significant interactions effects. Firstly, cancer diagnosis and the quality of healthcare people received in the past year were found to jointly affect their awareness of the goal of palliative care. Specifically, for people who had been diagnosed with cancer before, the quality of healthcare was positively associated with their awareness of palliative care. The better quality of healthcare they have received in the past, the more likely to believe the goal of palliative care to offer aid and support to patients. This might be explained by their higher trust in the medical system and healthcare providers due to their positive curative treatment experiences in the past, thereby possibly being more willing to hear about and discuss the palliative care option with their health care providers (2). They might even have received adequate palliative care referral and consultation in their healthcare facilities (14), which increased their awareness of palliative care. However, for people with no cancer diagnosis, quality of healthcare was not a significant influencing factor, which might be attributable to their relative lack of experience with hospital care or healthcare.

Secondly, for people who think they were in poor or moderate health condition, the level of perceived patient centeredness was positively related with their awareness of palliative care, which might be explained by their willingness to hear about and discuss the palliative care option with their health care providers due to their successful prior communication with them. Healthcare providers, particularly doctors, play a critical role in connecting patients with palliative care (14). They are mainly responsible for informing patients of treatment options, proposing referrals to palliative care, and arranging adequate treatment plans (54). When they believed that their emotional and psychological needs had been attended to in prior experiences, they might be more willing to learn about and discuss alternative treatment options with their health providers, thus having greater awareness of palliative care. Therefore, developing greater levels of perceived social support and past patient centeredness experience may substantially boost patient awareness of the positive goal of palliative care and their intentions to use palliative care. However, for people who think they were in excellent health condition, patient centeredness was not playing an influential role in influencing their awareness of the goal of palliative care, which might be explained by their less frequent communication with health providers and accordingly less likelihood to come across palliative care.

Interestingly, for people who think they are in poor health condition, patient centeredness is associated with greater awareness of palliative care but less favorable attitude (i.e., associating palliative care with giving up and death). This might be attributable to their overall lack of hope regarding illness recovery in their past treatment experiences and their heightened suspicion and negative beliefs about the effectiveness of palliative care. Johnson et al. (54) found that doctors considered it challenging to verbally refer palliative care option to patients and their caregivers because of the potential negative reactions (i.e., seen as equal to giving up). As suggested by the social ecological model (34), attitudes toward palliative care may be influenced by multiple levels of factors. Simply relying on doctor-patient communication to increase patients' attitudes and acceptance of palliative care might not be ideal, the synergic utilization of multiple levels of forces, including organizational, community and public policy factors, might be more effective and efficient in terms of improving public attitudes toward palliative care, particularly among people who think they are in poor health condition. Besides, for people who think they are in moderate or excellent health condition, the level of perceived patient centeredness is associated with more favorable attitude toward palliative care. This might be explained by the lower likelihood of triggered negative connotations and emotional damage given their better health condition. This yield important practical implications in terms of designing educational messages aiming at improving general public's attitude toward palliative care. Emphasis could be put on highlighting the positive patient centeredness experiences offered by palliative care.

While this study yielded several meaningful implications, it has several limitations that call for readers' caution in interpreting the findings and merit future research. First, the present study measured some variables (i.e., quality of health care received and perceived ability to take care of health) using single-item measures. Though some research suggests that carefully crafted single-item measures are as valid as multi-item measures of the same constructs (55), some other research posits that multi-item scales may outperform single-item scales in certain circumstances (56). As mixed results are presented, future research is needed to replicate our findings. Second, our data are descriptive rather than predictive, and our findings do not denote causal relationships between variables. In order to inform educational message design, future studies are needed to examine the causal relationships among personal and interpersonal factors, awareness of the goal of palliative care, and attitude toward palliative care. Finally, our results showed that female, non-white, and lower household income people reported greater awareness of palliative care, while female and more educated people showed more favorable attitude toward palliative care. It is worth conducting qualitative research to further examine why these groups had a favorable opinion toward palliative care. Understanding these may help to improve palliative care acceptance rates among patients who need it.

## Conclusion

Overall, this study tested how several personal and interpersonal factors were associated with individual awareness of and attitude toward palliative care. Findings identified several demographical disparities in awareness of and attitudes toward palliative care. In particular, female, non-white, and poorer people were more aware of the goal of palliative care, while female and more educated people had more favorable attitudes. In addition, interpersonal relationship quality was found to be a primary factor influencing people's awareness of and attitude toward palliative care. Moreover, cancer diagnosis history and perceived healthcare quality were found to jointly affect people's awareness of palliative care, while perceived health status and patient centeredness interacted to influence people awareness of and attitudes toward palliative care. For example, for people who think they are in better health condition, the level of perceived patient centeredness is associated with more favorable attitude toward palliative care. Our findings emphasize the importance of using precision messages and interventions to improve awareness of and attitude toward palliative care, and thereby boosting palliative care acceptance rate. Developing tailored palliative care educational resources for different audience segmentations can not only improve knowledge of the goal of palliative care but also help remove the barriers to adopting palliative care and ultimately increase palliative care acceptance rate.

## Data Availability Statement

Publicly available datasets were analyzed in this study. This data can be found at: https://hints.cancer.gov/data/download-data.aspx.

## Ethics Statement

The studies involving human participants were reviewed and approved by United States National Cancer Institute. The patients/participants provided their written informed consent to participate in this study.

## Author Contributions

XL and JL contributed to the conception and design of the study. XL performed the statistical analysis. All authors contributed to manuscript writing and revision, read, and approved the submitted version.

## Funding

This work was sponsored by Shanghai Pujiang Program.

## Conflict of Interest

The authors declare that the research was conducted in the absence of any commercial or financial relationships that could be construed as a potential conflict of interest.

## Publisher's Note

All claims expressed in this article are solely those of the authors and do not necessarily represent those of their affiliated organizations, or those of the publisher, the editors and the reviewers. Any product that may be evaluated in this article, or claim that may be made by its manufacturer, is not guaranteed or endorsed by the publisher.
